# Pharmacokinetics of combined gene therapy expressing constitutive human GM-CSF and hyperthermia-regulated human IL-12

**DOI:** 10.1186/1756-9966-32-5

**Published:** 2013-01-26

**Authors:** Fang Wei, Huiping Wang, Jufeng Zhang, Xiafang Chen, Chuanyuan Li, Qian Huang

**Affiliations:** 1 Experimental Research Center, First People’s Hospital, School of Medicine, Shanghai Jiaotong University, 85 Wujin Road, Shanghai, 200080, China; 2 Department of Dermatology, Duke University Medical Center, Durham, NC, 27710, USA

**Keywords:** GM-CSF, IL-12, Adenovirus, Hsp70, Hyperthermia

## Abstract

**Background:**

An adenovirus that expresses both interleukin (IL)-12 and granulocyte-macrophage colony-stimulating-factor (GM-CSF) has been proven to be very effective in treating several tumors, but causes serious normal tissue toxicities.

**Methods:**

In this study, a novel adenoviral vector was constructed by placing the human GM-CSF gene under the control of the CMV-IE promoter and human IL-12 gene under the control of heat shock protein 70B gene promoter. Both hGM-CSF and hIL-12 expressions in virus-infected tumor cells were analyzed *in vitro* and *in vivo* when underlying single or multiple rounds of hyperthermia.

**Results:**

We observed constitutive high expression of human GM-CSF and heat-induced expression of human IL-12 after a single round of hyperthermia post viral infection. The heat-induced hIL-12 expression exhibited a pulse-like pattern with a peak at 24 hrs followed by a decline 48 hrs post heat stress. Repeated heat treatment was more effective in inducing hIL-12 expression than a one-time heat treatment. Interestedly, we also observed that constitutive expression of hGM-CSF could be stimulated by heat stress in tested tumor cells.

**Conclusion:**

Our study provided a novel strategy for combined gene therapy that allows constitutive expression of a non-toxic gene such as GM-CSF and heat-induced expression of a toxic gene such as IL-12. In addition, our study also showed that hyperthermia can be used to trigger gene expression in temporal and special manner.

## Introduction

The unique ability of cancer to exploit the immune system in order to promote tumor growth and suppress immune response makes cancer therapy difficult. However, modulation of the immune system should provide promising results. Cytokines are a large family of intercellular signaling peptides that function in the regulation of immune response. Cytokine therapy has been reported to be an effective strategy at inducing strong antitumor immune response [1]. However, initial studies using systemic treatment with recombinant cytokines produced discouraging results due to dose-limiting toxicities [2]. Compared to protein therapy, gene therapy proved to be as efficient while inducing less toxicity [3]. Among these cytokine-based gene therapies, an adenovirus that expresses both interleukin (IL)-12 and granulocyte-macrophage colony-stimulating-factor (GM-CSF) has been proven to be very effective in treating several tumors [4,5]. However, current adenoviruses deliver constitutive IL-12 gene expression, which causes serious normal tissue toxicity [6].

GM-CSF is a growth factor capable of enhancing antitumor activity by activating dendritic cells (DCs) to improve antigen presentation. GM-CSF can also activate macrophages and induce the release of tumor necrosis factor (TNF) [7] to achieve an antitumor effect. In addition, GM-CSF can indirectly stimulate T-cell activation via interleukin-1 release [8]. However, increased cellular GM-CSF expression also leads to counter-regulatory immune responses to decrease the expansion of cytotoxic T cells (Tc), thereby limiting its antitumor activity [7]. In contrast, IL-12 has been shown to exert potent immunostimulatory effects on certain helper T cells as well as cytotoxic T lymphocytes (CTL) and natural killer (NK) cells [9]. Therefore, the combined use of GM-CSF and IL-12 can counteract the counter-regulatory role of GM-CSF on Tc and increase the immune benefits of GM-CSF.

Human IL-12 is a disulfide-linked heterodimer composed of 35 and 40 kDa subunits. Preclinical studies and clinical trials of IL-12 gene therapy showed that this treatment can induce remarkable anti-tumor response in various tumors, including melanoma, sarcoma, and adenocarcinoma [3]. However, both preclinical and clinical tests revealed that IL-12 gene therapy can induce highly toxic side effects [3]. This is because high constitutive IL-12 expression increases IFN-γ production [10]. Thus, IL-12 expression in gene therapy requires regulation. However, the current adenovirus coexpressing GM-CSF and IL-12 genes does not account for the regulation of IL-12. Heat-based gene regulation is a ubiquitous stress response to heat shock in mammalian cells. Based on this feature, heat shock protein 70 promoter (hsp70B) has been widely used in gene therapy to control gene expression [6]. The pharmacokinetics of GM-CSF and IL-12 production as well as possible interactions between constitutive GM-CSF expression and heat-induced IL-12 expression should be investigated before clinical use. However, there is the dilemma that IL-12 has a restrict species-specificity. For example, human IL-12 shows no activity in animal models and mouse IL-12 has no activity in human. Although the efficacy and toxicity of sustained human IL-12 expression cannot be evaluated in an animal model, the expression pattern of the adenoviral vector must be first tested in an animal model before entering clinical trials.

Currently, gene therapy with combined GM-CSF and IL-12 has been established in several kinds of tumors using adenovirus to express constitutive GM-CSF and IL-12 levels. Although significant anti-tumor effect has been observed, the level of GM-CSF or IL-12 is not regulatory [4,11]. In this study, we first constructed a novel adenoviral vector that allowed constitutive expression of human GM-CSF and heat-induced expression of human IL-12. The pharmacokinetics of gene expression triggered by hyperthermia was then tested in cell culture and in an animal model. Our study provided insights on tumor therapy by combining gene therapy with hyperthermia.

## Materials and methods

### Cell culture

A549, a human non-small cell lung carcinoma cell line, and Hep3B, a human hepatoma cell line, were purchased from American Type Culture Collection. All cells were cultured in RPMI 1640 with 10% fetal bovine serum, 100 units/mL penicillin, and 100 μg/mL streptomycin at 37°C, 5% CO_2_.

### Adenovirus preparation

**Figure 1:**
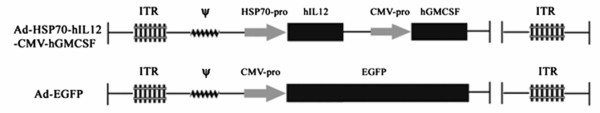
**A schematic diagram of adenovirus used in this study.** HSP70-pro: heat shock protein 70 gene promoter; hIL12: human interleukin 12; CMV-pro: CMV promoter; hGMCSF: granulocyte-macrophage colony-stimulating-factor gene; EGFP: enhanced GFP.

The adenovirus used to establish constitutively high expression of human GM-CSF and heat-inducible expression of human IL-12 was constructed according to established protocols [12] using commercially available plasmids (Microbix, Toronto, Canada). To construct the heat-inducible IL-12 expression cassette, cDNAs for both the p40 and p35 subunits of human IL-12 were inserted into the E1 region under control of the human *hsp70B* gene promoter [13,14]. The p40 and p35 subunits were connected using an internal ribosome entry site sequence [15] so that both subunits could be transcribed under the control of the same promoter. The human GM-CSF expression cassette was constructed by placing the human GM-CSF gene under the control of a constitutively active CMV-IE promoter in the E1 region [16] (see Figure 1). The completed adenovirus called Adcmv-GMCSF-HSP-IL12 will establish constitutive expression of human GM-CSF and heat-inducible expression of human IL-12. Large scale preparation of recombinant Adcmv-GMCSF-HSP-IL12 was accomplished as previously described [17]. The control vector is an adenovirus expressing GFP protein (Figure 1).

### In vitro heating experiments

A549 and Hep3B cells were seeded in 24-well plates at a density of 6 × 10^4^ cells/well. After cells were cultured for 24 hrs, 100, 500, and 1000vp (viral particles) of Adcmv-hGMCSF-hsp-hIL12 virus were added into each well. Twenty-four hours later, the culture medium was replaced with 1 ml of fresh medium containing 2% FCS and cells were heated in a 45°C water bath for 45 min. Twenty-four hours later, the medium was collected for hGM-CSF and hIL-12 measurement and replaced with 1 ml of fresh medium. Cells were heated again (45°C, 45 min) and the medium was collected 24 hrs post heating.

### In vivo heating experiments

Balb/C nude mice (BALB/c, *nu/nu*) weighing 20-22 g were provided by the animal center of Shanghai Biological Science Institution and housed in rooms under standard lighting conditions and temperature. Water and food were provided *ad libitum*. All animal experiments were conducted under an approved protocol from Shanghai Jiaotong University and performed in accordance with the animal care guidelines of the Chinese Council. Hep3B tumors were introduced by subcutaneous injection of 1 × 10^7^ Hep3B cells in 50 μL of PBS into the right hind limbs of mice. When tumor size reached 1 cm in diameter, a total of 2 × 10^8^ Adcmv-hGMCSF-hsp-hIL12 was injected into tumor. Mice were divided into 3 groups: non-heating group, one-time heating group, and three-time heating group. In non-heating group, animals were sacrificed on day 1, 2, 3 and 4 post virus injection. In the one-time heating group, tumors were heated once 24 hrs post virus injection and animals were sacrificed on day 1, 2, 3 and 4 post heat treatment. In three-time heating group, tumors were heated on day 1, 3, and 5 post virus injection and animals were sacrificed on day 4, 5, 6, 7 post first heat treatment. Tumors were heated to 42°C in a water bath for 40 min by immersing the tumor-bearing leg in the water bath [18]. Tumor tissues were homogenized for hGM-CSF and hIL-12 detection.

### Detection of GM-CSF and IL-12 levels

The hGM-CSF and hIL-12 levels in cell culture medium and tumor tissues homogenate were detected with human GM-CSF and human IL-12 ELISA kits (R&D Systems, Minneapolis, MN).

## Results

### hGM-CSF and hIL-12 expression in Adcmv-hGMCSF-hsp-hIL12 virus infected A549 and Hep3B cells

**Figure 2:**
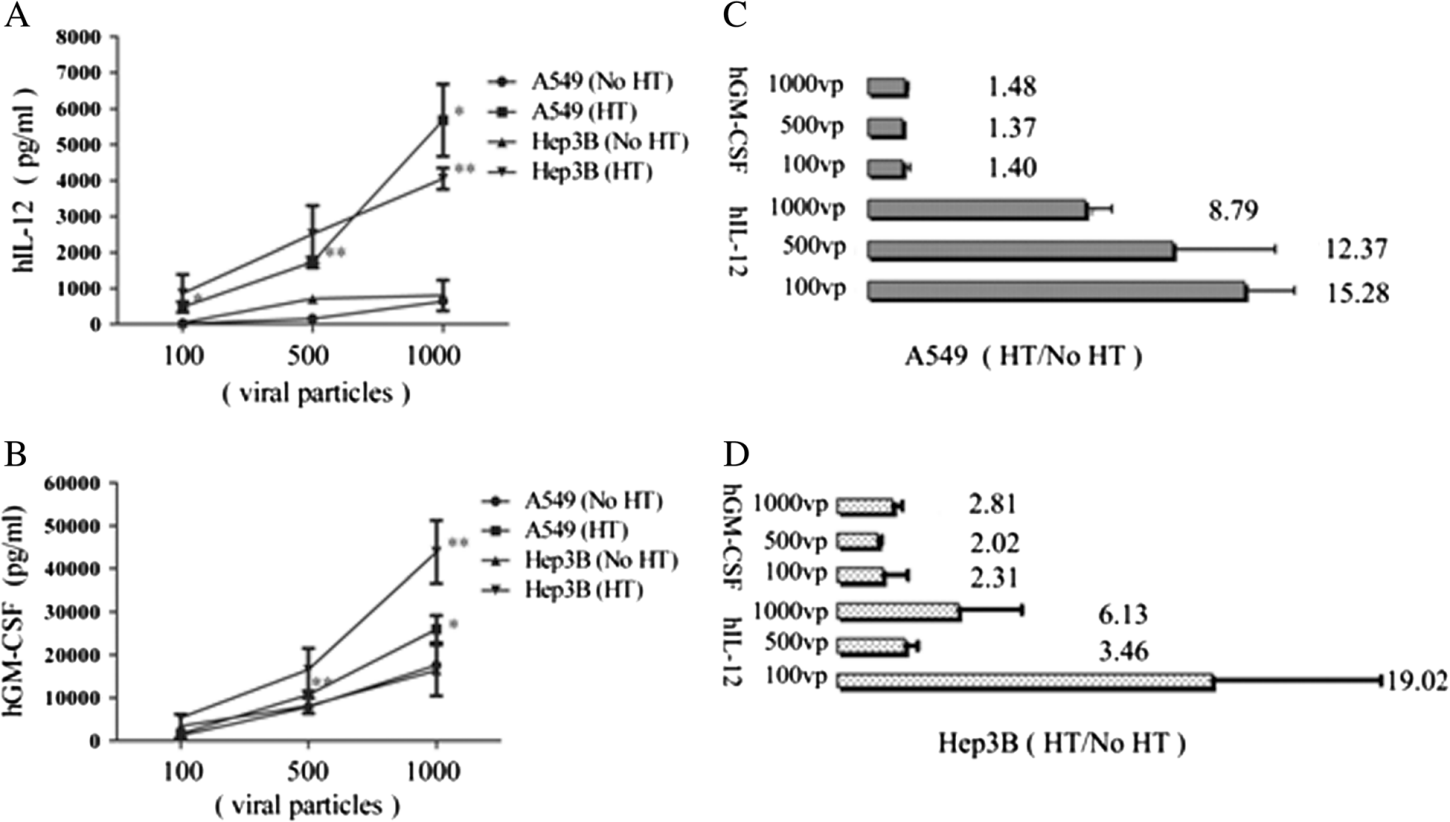
**hGM-CSF and hIL-12 expression in heat treated A549 and Hep3B cells.** A549 and Hep3B cells in 24-well plates were infected with Adcmv-hGMCSF-hsp-hIL12 virus for 24 hrs and heated at 45°C for 45 min. Twenty-four hours late, medium was collected for hGM-CSF and hIL-12 measurement. **A**) hIL-12 expression under heating and no heating treatment. **B**) hGM-CSF expression under heating and no heating treatment. **C**) Relative hGM-CSF and hIL-12 expression in A549 cells. **D**) Relative hGM-CSF and hIL-12 expression in Hep3B cells. HT: heating treatment. N = 5 repeated experiments.

As shown in Figure 2, 1000, 500 and 100 viral particle per cell (vp) infected cells exhibited significant increases in the production of hGM-CSF and hIL-12 in A549 after heat treatment (Figure 2A, B). In Hep3B cell medium, 1000 vp of virus infection significantly increased hIL-12 (*p*=0.001) and hGM-CSF (*p* = 0.008) production 24 hrs after heat treatment. 500 vp and 100 vp virus infected cells also exhibited significant increases in the production of hGM-CSF and hIL-12 after heat treatment (Figure 2A, B). Heat treatment induced 8.79 ± 0.64 and 12.37 ± 2.41 fold increases in hIL-12 production in 1000 vp and 500 vp virus infected A549 cells (Figure 2C). In Hep3B cells, heat treatment induced 6.13 ± 1.89 and 3.46 ± 0.36 fold increases in cells infected with 1000 vp and 500 vp virus respectively, whereas heat treatment induced 19.02 ± 4.95 fold increase in cells infected with 100 vp virus (Figure 2D). In both A549 and Hep3B cells, hGM-CSF expression showed dependence on virus dosage. Although hGM-CSF was driven by CMV promoter, hGM-CSF expression was increased 1.48 ± 0.08 fold in A549 cells and 2.81 ± 0.29 fold in HepB3 cells after heat treatment.

### The effect of heat treatments on hGM-CSF and hIL-12 expression

**Figure 3:**
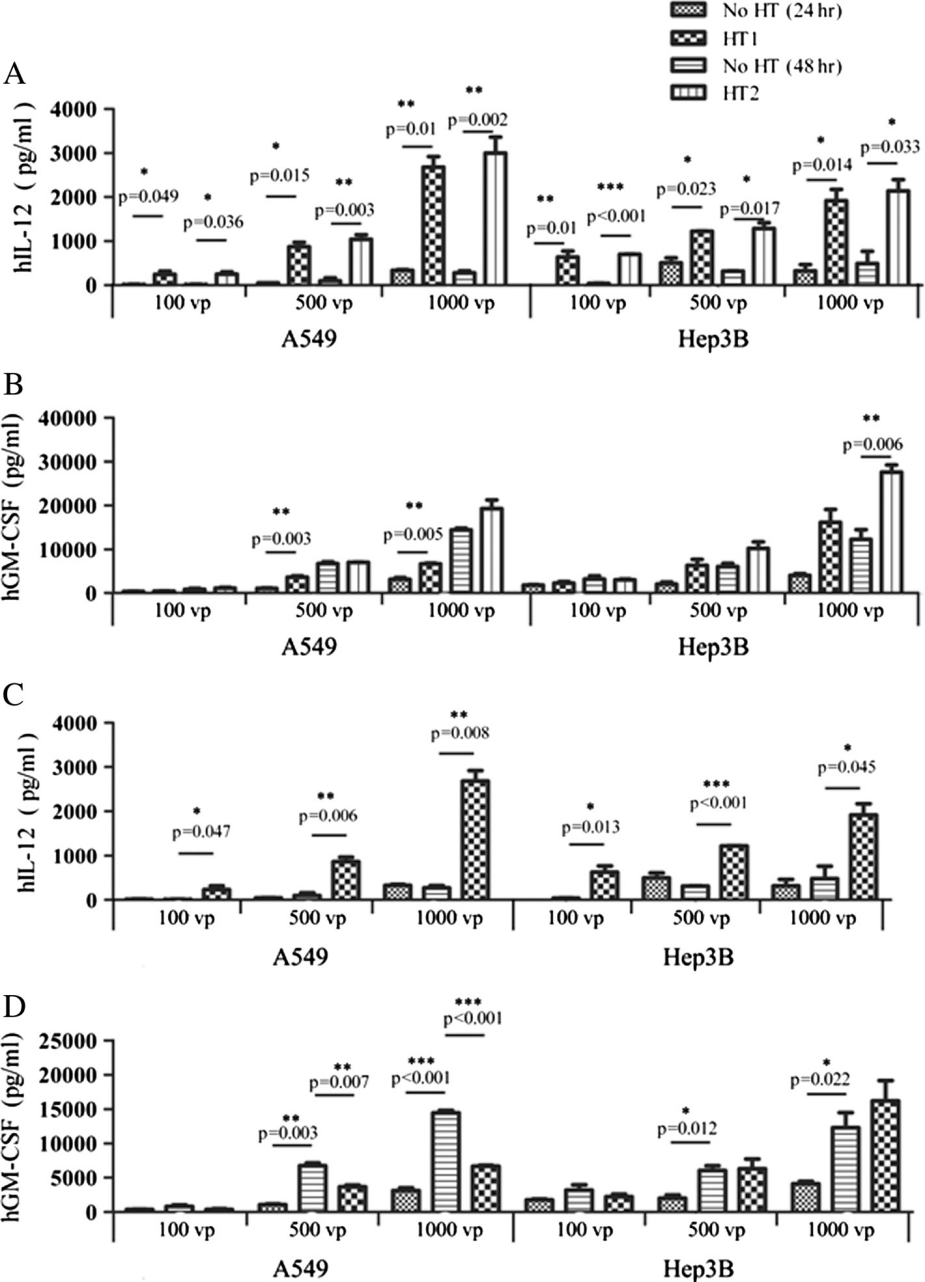
**The time dependence of hGM-CSF and hIL-12 expression in heat treated A549 and Hep3B cells.** Cells were infected and heated as described in Figure 2. Medium was collected at 24 and 48 hrs after heating treatment. **A**) hIL-12 expression in A549 and Hep3B cells. **B**) hGM-CSF expression in A549 and Hep3b cells. **C**) Comparison of hIL-12 expression between cells heated for 24 hrs and cells without heating for 24 and 48 hrs. **D**) Comparison of hGM-CSF expression between cells heated for 24 hrs and cells without heating for 24 and 48 hrs. N = 5 repeated experiments.

As shown in Figure 3A in non-heated A549 cells, first heat treatment significantly increased hIL-12 levels in A549 cells infected with 100 vp 500 vp, 1000 vp virus, respectively, while the second heat treatment was more efficient in increasing hIL-12 levels in A549 cells *(p* < 0.05 at all 3 viral dosages). In non-heat treated Hep3B cells, first heat treatment significantly increased hIL-12 expressions in Hep3B cells 24 hrs after first heat treatment. The second heat treatment was also more efficient in increasing hIL-12 levels in Hep3B *(p* < 0.05 at all 3 viral dosages). These results suggest that hIL-12 expression is heat-inducible. In contrast, first heat treatment significantly increased hGM-CSF expression in A549 cells infected with 500 vp and 1000 vp virus in non-heat treated A549 cells shown in Figure 3B; however, second heat treatment did not significantly increase hGM-CSF expression in A549 cells (*p* > 0.05). In non-heat treated Hep3B cells, first heat treatment increased hGM-CSF levels in Hep3B cells but showed no statistical difference (*p* > 0.05). After second heat treatment, significant difference was observed in Hep3B cells infected with 1000 vp virus. These results suggest that heat treatment can increase hGM-CSF expression, but hGM-CSF expression is not heat-dependent.

We further compared the expression of hIL-12 (Figure 3C) and hGM-CSF (Figure 3D) in A549 and Hep3B cells infected with the virus underlying heat treatment for 24 hrs and no heat treatment for 24 and 48 hrs. Results showed that there were no significant differences in hIL-12 levels between 24 and 48 hrs in both A549 and Hep3B cells infected with 3 different viral doses underlying no heat treatment, but a significant increase in A549 and Hep3B cells was observed after 24 hrs of heat treatment. These results suggest that hIL-12 expression is heat-inducible, but not time-dependent. In contrast, significant differences in hGM-CSF levels were observed in A549 and Hep3B cells infected with 500 vp and 1000 vp virus underlying no heat treatment for 24 and 48 hrs. In A549 cells, heat treatment for 24 hrs increased hGM-CSF levels, but hGM-CSF levels were actually lower than in non-heat treated A549 cells for 48 hrs. In Hep3B cells, heat treatment for 24 hrs increased hGM-CSF levels, but hGM-CSF levels were equal to or higher than in non-heat treated Hep3B cells for 48 hrs. These results suggest that hGM-CSF expression is time-dependent but not heat-dependent.

### The effect of heat treatment on in vivo hGM-CSF and hIL12 expression

**Figure 4:**
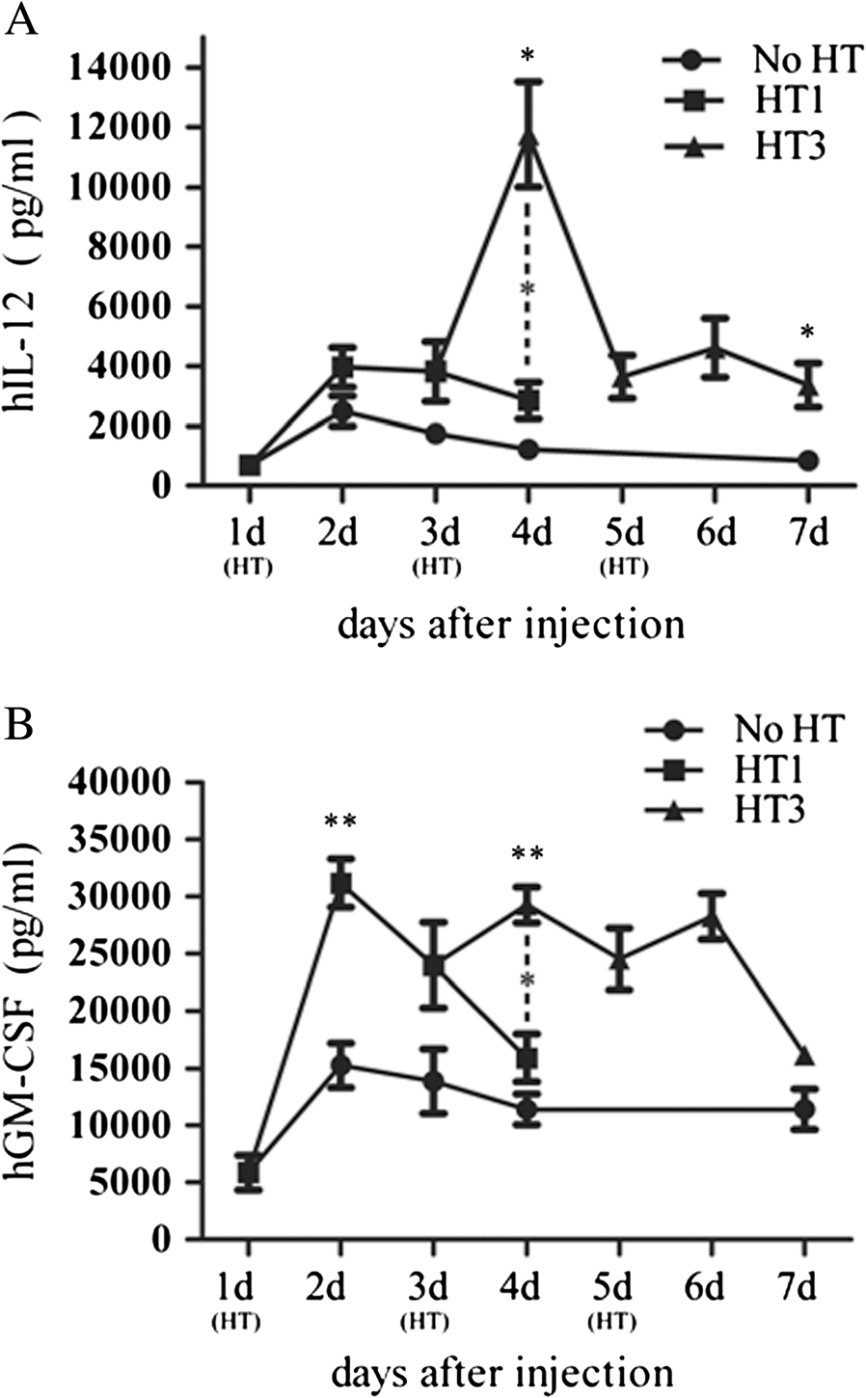
**hGM-CSF and hIL-12 expression in Hep3B tumor tissues.** Adcmv-GMCSF-hsp-hIL12 was intratumorly injected. Tumors were not heated, heated for 1 time, 2 time, and 3 times at 42°C for 40 min. Animals were sacrificed at different time point and tumor tissues were homogenized for hGM-CSF and hIL12 detection. **A**) hIL-12 expression in tumor tissues. **B**) hGM-CSF expression in tumor tissues. N = 5 mice per group.

As shown in Figure 4, virus infection produced consistent hGM-CSF and hIL-12 expression under no heat treatment. hGM-CSF expression was significantly higher than hIL-12, but both reached their peak at 24 hrs after virus infection and began to decline slowly at 48 hrs post virus infection until day 7 of our observation. Under heat treatment, hIL-12 and hGM-CSF expressions were significantly increased and reached a peak at 24 hrs after each heating and began to decline 48hrs after heating.

As shown in Figure 4A, intratumoral injection of adenoviral vectors led to lower IL-12 expression. The first heat treatment elevated hIL-12 level from 2500 ± 506 pg/ml (no HT) to 3966 ± 661 pg/ml (*p* = 0.207), but second heat treatment induced 9.53 fold increase in hIL-12 expression compared to no heat treatment (*p* = 0.034) and 4.1 fold increase compared to first heat treatment (HT1) (*p* = 0.036). Although the third heat treatment (HT3) was less effective than the second heat treatment, hIL-12 level was still higher in heat treated tumors than in non-heat treated tumors on day 7 since first treatment (*p* = 0.039), suggesting that multiple heat treatments could keep a constitutively low hIL-12 expression with a peak-like expression at 24 hrs after heating.

As shown in Figure 4B, the expression of hGM-CSF was controlled by CMV promoter; however, hGM-CSF expression in tumor tissues increased 2.04 fold (*p* = 0.009) after first heat treatment compared to non-heat treated tumor tissues (*p* = 0.013). The expression of hGM-CSF increased in tumor tissues within 24 hours after 2nd (*p* = 0.002) and third (*p* = 0.013) heat treatments. However, the peak concentrations of hCM-GSF after heating were similar, and no significant difference was observed between first, second, and third heating treatments.

## Discussion

Combined gene delivery has been widely adopted in gene therapy to increase therapeutic efficacy. However, some gene products are very toxic to normal tissues, which limit effective clinical application. To overcome this obstacle, the expression of one or more genes in the combined delivery should be regulated. Gene therapy utilizing a combination of IL-12 and GM-CSF has been previously established [4,5]. In this strategy, GM-CSF is able to stimulate proliferation, maturation, and function of antigen representation cells, while IL-12 is able to enhance T-helper 1 cell’s immunity, increase cytotoxicity of T-lymphocytes, and inhibit angiogenesis [5,8]. Another benefit of this strategy is that IL-12 can counteract the negative regulation of GM-CSF on Tc cells [7]. However, high toxicity was observed with this combination due to the consistently high IL-12 expression. To overcome the high toxicity, we constructed an adenovirus to constitutively express human GM-CSF while controlling IL-12 expression via a heat-inducible promoter. After viral infection, heat stress induced a pulse-like expression of hIL-12 and a high constitutive expression of hGM-CSF *in vitro* and *in vivo*.

Consistent with previous reports, constitutive hIL-12 expression was very low in both the A549 and Hep3B cells under no heating. Heat stress induced 15 to 19 fold increases in hIL-12 expression in cultured cells, while it induced a 16.9 fold increase in Hep3B tumor tissues after a second heat treatment. This suggests that hsp70 promoter is highly inducible with low background activity. Consistent with our previous findings, heat-induced hIL-12 expression peaked at 24 hrs and began to decline at 48 hrs post heat treatment [18]. This pattern can reduce the consistently high IL-12 expression-induced toxicity. In addition, we found that the second heat treatment is more effective than the first heat treatment in inducing hIL-12 expression, but the third heat treatment is less effective than the second heat treatment. The lower efficacy of the third heat treatment in inducing gene expression may suggest that one injection of non-replicating adenovirus can only support a limited number of heat treatments that induce gene expression. In addition, high virus dose could produce high hIL-12 expression under heat stress. However, low dose infection produced relatively higher amplification rate in hIL-12 expression due to the existence of low leak in hsp promoter activity. This observation supports the idea that the virus dose can be selected for clinical application. We acknowledge that we didn’t test the temperature-dependent effect of IL-12 expression and that is a weakness to this study. However, previous studies demonstrated a temperature-dependent effect in hsp70 promoter controlled gene expression [19,20]. The second weakness is that the activity and toxicity of inducible human IL-12 cannot be tested in the animal model because human IL-12 shows no activity in animals and the nude mice used in this study are immunodeficient.

In this study, the adenovirus was constructed with a CMV-IE promoter to control human GM-CSF expression. The CMV promoter should produce highly constitutive hGM-CSF expression. However, heat treatment at 45°C increased hGM-CSF expression by 1-1.5 folds in A549 cells and 2-3 folds in Hep3B cells. In addition, the *in vivo* heating study revealed that although the first heat treatment induced a 2-fold increase in hGM-CSF expression, the second and third heat treatments didn’t increase hGM-CSF expression as much as the first treatment. In contrast, hGM-CSF expression was stable for 6 days after first heat treatment and declined on day 7. This observation suggests that the heat-inducible hGM-CSF expression is not heat-dependent but time-dependent. We also noted that heat induction of hGM-CSF expression is more obvious in Hep3B cells than in A549 cells, suggesting cell type dependence. Recently, the stimulating effect of heat stress on CMV promoter activity has been studied [21,22]. Although the possible mechanisms might be complex, a considerable homology to the heat stress element core consensus (GA–TCC) within 18 bp elements in IE enhancer might be the most reasonable explanation [21,23]. Heat stress might regulate CMV-IE activity directly and indirectly through heat-activated transcription factors. Heat stress inducing various transcriptional factors, including those activating the CMV-IE promoter, has been reported [21,22]. Therefore, the cell type dependence might reflect the high specificity of the signaling pathway and transcription factors.

In this study, we established constitutive high expression of human GM-CSF and heat-induced expression of human IL-12 with a single adenoviral vector. The heat-induced hIL-12 expression has a pulse like shape with a peak at 24 hrs post heat stress that is maintained for 24 hrs in tumor tissues. Repeated heat treatments are effective but limited by the clearance of non-replicating adenovirus. Together with the low background activity of hsp70 promoter, heat induced gene expression enables a fairly strict control of gene expression, which diminishes the cytotoxicity of toxic cytokines . We also observed that the CMV-IE promoter driven constitutive high expression of hGM-CSF could be stimulated by heat stress in a cell type dependent manner. However, the CMV-IE promoter activity cannot be regulated by heat stress. Our study provided solid evidence for the feasibility of heat-induced regulation of gene expression in a combined gene delivery vector.

## Competing interests

All authors declared no any conflict of interest.

## Authors’ contribution

FW: Conduct experiments, prepare manuscript HW: perform experiment, data analysis JZ: perform experiments XC: cell culture CL: experiment design, manuscript revision QH: experiment design, final approval of manuscript. All authors read and approved the final manuscript.
